# A decade of vertebrate palaeontology research: global taxa distribution, gender dynamics and evolving methodologies

**DOI:** 10.1098/rsos.250263

**Published:** 2025-05-14

**Authors:** Haohan Wang, Juliana Sterli, Vincent Dupret, Henning Blom, Annalisa Berta, Susan Turner, Daoming Han, Luyan Xu, Zhaohui Pan

**Affiliations:** ^1^ Research Center of Natural History and Culture, Qujing Normal University, Qujing, Yunnan, People’s Republic of China; ^2^ Key Laboratory of Yunnan Provincial Department of Education of the Deep-Time Evolution on Biodiversity from the Origin of the Pearl River, Qujing Normal University, Qujing, Yunnan, People’s Republic of China; ^3^ Consejo Nacional de Investigaciones Científicas y Técnicas (CONICET), Museo Paleontológico Egidio Feruglio (MEF), Trelew, Argentina; ^4^ Department of Organismal Biology, Uppsala University, Uppsala, Sweden; ^5^ Department of Biology, San Diego State University, San Diego, CA, USA; ^6^ Queensland Museum Geosciences, Hendra, Australia; ^7^ School of Geography and Tourism, Qujing Normal University, Qujing, Yunnan, People’s Republic of China; ^8^ Institute of Vertebrate Paleontology and Paleoanthropology, Chinese Academy of Sciences, Beijing, People’s Republic of China

**Keywords:** vertebrate palaeontology, bibliometric analysis, gender gap, methodological evolution, latent Dirichlet allocation topic modelling

## Abstract

Using 12 104 publications from 2014 to 2023 in the DeepBone database, this study employs bibliometric methods, including full-text latent Dirichlet allocation (LDA) modelling, co-occurrence network analysis and geographic mapping with ArcGIS, to examine three key aspects of vertebrate palaeontology development: geographic distribution of newly established taxa, gender demographics among researchers and research trends. Gender data were analysed using automated tools with manual verification to ensure accuracy, while methodological evolution was investigated through systematic text mining and classification. Among 8336 newly established taxa, mammals (34.72%) and fishes (29.76%) dominate, followed by reptiles (25.34%), birds (7.39%) and amphibians (2.80%). Geographic analysis reveals significant regional disparities, with the USA (13.50%) and China (13.32%) contributing the most, while Africa and Oceania remain under-represented (less than 10%). Gender analysis indicates a gradual increase in female representation from 22.78 to 27.20% over the decade, highlighting the imperative to address gender disparities in vertebrate palaeontology, thereby advancing equity in alignment with UNESCO Sustainable Development Goal 5. LDA topic modelling identifies 15 distinct research topics, encompassing evolutionary biology, cranial and skeletal morphology, dinosaur–bird evolution and human evolution, while co-occurrence analysis highlights the evolution of research methodologies, revealing strong interconnections between phylogenetic analysis (15%), traditional morphological analysis (12%) and high-resolution imaging techniques (9%).

## 1. Introduction

Building upon advancements in prior decades, vertebrate palaeontology has witnessed particularly significant progress in the last 10 years, driven by the emergence of new analytical methods and research paradigms [1–3]. A comprehensive analysis of the current state of vertebrate palaeontology research is essential for understanding the field’s development trajectory and research priorities. The rapid advancement of bibliometrics and big data science offers reliable methods for systematically, quantitatively and objectively analysing the contributions of key authors and institutions, their collaborative relationships and for summarizing research frontiers, hotspots and potential issues [4]. While bibliometric analyses have been widely applied in various scientific disciplines, including geoscience [5], there remains a paucity of such studies specifically focused on vertebrate palaeontology. Notably, our previous study quantitatively analysed the national and institutional collaboration networks, as well as the characteristics of disciplinary development and evolution in vertebrate palaeontology from 2013 to 2022, using bibliometric methods [6]. This work deepened scholars’ understanding of global developments in the field.

Traditional palaeontological research aims to understand past organisms and locate them in time, space and within the evolutionary tree. To achieve this goal, palaeontological research focuses primarily on three core aspects. First, it relies on detailed morphological descriptions of fossil specimens. Second, it involves comprehensive phylogenetic analyses. Third, it incorporates crucial contextual information such as lithostratigraphy, taphonomy, palaeoecology, palaeoenvironment and palaeogeography. However, with the emergence of increasingly large-scale data-driven palaeontological studies, the use of quantitative methods and data-driven research models has become the norm in life and earth sciences [7]. This development in research approaches necessitates not only studies on spatial distribution patterns of new taxa, which can reflect the development trends of traditional vertebrate palaeontology research [8], but also the understanding of how research methodologies themselves have evolved and become integrated over time.

Women are under-represented in palaeontology [9–11]. Two decades after the largest professional organization of vertebrate palaeontologists—the Society of Vertebrate Paleontology (SVP)—was founded in 1940, women made up less than 10% of the membership [12]. Now women SVP members comprise 36% with the greatest growth among student members. Despite this growth, less than 25% of members have jobs as professors or curators [12]. Eliminating gender gaps in academia can reduce biases and discrimination in academic careers, diversifying science, and is a critical step towards achieving academic and technological breakthroughs [13]. Recent studies have highlighted the severity of this issue, with nearly half of researchers leaving academia within a decade of their first publication, particularly among female scientists [14]. This aligns with the Organisation for Economic Co-operation and Development (OECD) findings indicating that gender gaps in science, technology, engineering and mathematics (STEM) fields persist globally, with women under-represented in advanced scientific roles [15–18], in editorial boards of scientific journals [19], in the news [20,21], in online dissemination of scientific works [22], among others. Studies across different geographical and economic contexts reinforce these patterns. For example, cross-national analyses in both developed and developing regions show that despite women’s increasing participation and often superior academic performance in STEM fields [23,24], their representation in advanced academic positions remains consistently low, particularly in research leadership roles. These disparities are often attributed to structural and societal barriers, such as high childcare costs and limited career support for women [15]. This concerning trend underscores the urgent need to understand and address gender dynamics within specific scientific disciplines. Despite these critical considerations, little was known about the gender dynamics within the vertebrate palaeontology research community until a recent study [12,25], although this topic has been more intensively addressed within the larger palaeontological community [9–11,26]. Therefore, understanding the dynamic changes in gender proportions is of great significance for recognizing the progress of gender equality in vertebrate palaeontology research, promoting gender equality and diversity, and creating a more inclusive academic environment.

Topic modelling is one of the most powerful techniques for uncovering latent data relationships within texts. Latent Dirichlet allocation (LDA), as a topic modelling tool, has been widely used in text mining and research trend analysis [27]. Although our previous study conducted a preliminary analysis using LDA modelling on abstracts, it did not delve into full-text analyses or explore the deeper thematic structures of the field [6]. A comprehensive full-text analysis is hence needed to reveal deeper insights into research trends.

The completeness and accuracy of reference data are vital for ensuring that bibliometric research objectively reflects the development characteristics of a discipline. To support comprehensive analyses, particularly full-text LDA modelling, a reliable and extensive database is essential. In 2019, the DeepBone database (www.DeepBone.org) was initiated to address this need, aiming to create the most comprehensive and high-quality database of vertebrate palaeontology on the Internet. Over the years, the DeepBone database has developed into a specialized database dedicated to vertebrate palaeontology, providing structured specimen-based data and associated bibliographic information. This specialized organization of data enables systematic analyses of research patterns and trends [28].

While our previous study provided initial insights on research trends and academic hubs based on abstract analysis, significant knowledge gaps remained in understanding the holistic scope of research evolution in vertebrate palaeontology [6]. To address these gaps, we have completed the full-text processing and analysis of 12 104 vertebrate palaeontology research papers from 2014 to 2023 through the DeepBone project. Through this comprehensive dataset, we aim to address in this new work three critical aspects of the field’s development: (i) the global distribution patterns of newly added taxonomic units, revealing geographical hotspots and taxonomic trends, which will help identify productive research regions and underexplored areas; (ii) the changing dynamics of gender representation in the research community, providing insights into progress towards academic equality and areas needing improvement; and (iii) the thematic structure and methodological evolution of the discipline, examined through both full-text LDA analysis of research topics and co-occurrence network analysis of research approaches, offering deeper insights into how vertebrate palaeontology has developed as a scientific field over the past decade. By combining these analyses, the present study not only provides a deeper understanding of the current status and future trends in vertebrate palaeontology research but also offers valuable insights for researchers, institutions and funding agencies in strategic planning and resource allocation. We also hope this study will contribute to achieving gender equality, aligning with UNESCO’s Sustainable Development Goal 5 (https://www.unesco.org/en/sdgs).

## 2. Material and methods

### 2.1. Data collection

The primary data for this study were sourced from the DeepBone database, updated as of 1 September 2024. Building upon the literature data used in Wang *et al*. [6], which covers 2013–2022, we shifted the dataset to cover the full 2014−2023 time period [6], focusing on global vertebrate palaeontology research articles, with selection criteria and categorization methods detailed therein. This resulted in a total of 12 104 articles for analysis. To prepare the articles for full-text LDA analysis [29], the PDF full texts were converted to TXT format via optical character recognition (OCR). After OCR processing, each article was manually checked against the original PDF. This involved line-by-line comparison, meticulously performed by our data entry team, to identify and correct any discrepancies or errors introduced during OCR. All articles were individually searched in the Web of Science Core Collection (WOS) using their titles as search terms. The bibliographic information retrieved was exported in ‘plain text file’ format with ‘full record and cited references’ for gender analysis software recognition and information extraction [30]. A total of 12 035 bibliographic records were retrieved and exported from WOS, accounting for 99.43% of the total number of articles. The remaining 0.57% could not be included as they were not indexed in WOS at the time of our data export, which limited their availability for gender analysis.

### 2.2. Taxonomic remark

As in our former article, choices had to be made regarding larger categories of taxonomic units and systematic content, especially for evolutionary transitional forms. Thus, fishes contain taxa such as *Tiktaalik*, and feathered dinosaurs are categorized as reptiles rather than birds [6].

### 2.3. Geographic distribution analysis

Since some research papers deal with fossil materials from multiple countries, we manually compiled the collection locations from where only the type specimens came from for new taxonomic units, ensuring consistency by categorizing regions according to ISO 3166-1 alpha-2 standards [31]. This type of specimen-based approach allows us to map the geographic distribution of newly described taxa over the past decade. Using ArcGIS Pro 3.0 software, we plotted a frequency map to visualize the density and distribution of research areas in global vertebrate palaeontology over the past decade. Based on the number of new taxonomic units defined in different regions, we analysed the hotspot areas of vertebrate palaeontology research. The base map was derived from the 1 : 41 million world map digital vectorization provided by the National Bureau of Surveying and Mapping Geographic Information of China (http://bzdt.ch.mnr.gov.cn/). National administrative divisions were executed according to the ISO 3166-1 alpha-2 international standard.

### 2.4. Gender analysis

Based on the bibliographic information exported from WOS, we extracted all author information from the C1 field and the first author information in the standard author sequence (hereinafter referred to as the first author), as well as extracted the corresponding author information from the Reprint Address (RP) field. Author information includes first name, family name, affiliation and country. We selected the Genderize online tool (https://genderize.io/) for its high accuracy and broad acceptance among leading academic institutions and organizations in gender-based analyses [30]. The use of the Genderize tool in our study was a compromise to prioritize the safety and privacy of individuals who may not be able or willing to disclose their gender information publicly. This approach allows us to analyse available data while acknowledging the inherent risks and limitations associated with gender disclosure. This tool is trusted by institutions like Columbia University and has been recognized in both peer-reviewed scientific literature (*Nature*) and public science communication (*The Guardian*), demonstrating its wide acceptance across different platforms while ensuring methodological rigour and data consistency. All authors, first authors and corresponding authors were uploaded to the Genderize online tool for gender identification. For authors with a gender probability accuracy lower than 0.8, manual verification was conducted (via telephone inquiries and institutional website searches). Finally, we used R 4.3.2 [32] to analyse the changing trends in gender proportions among all authors, first authors and corresponding authors in vertebrate palaeontology research over the past decade. Building on these data, we further analysed the gender distribution in global vertebrate palaeontology research in 2023, as well as gender proportion trends among the top five countries by publication volume over the last 10 years [6]. Additionally, we examined gender ratio dynamics across different vertebrate groups studied over the same period, distinguishing between the proportion of authors by gender and the proportion of publications produced by authors of different genders.

### 2.5. Topic modelling

To ensure consistency and relevance, we pre-processed the literature data through several steps: removing non-alphabetic characters, converting all text to lower case and eliminating common stop words from the Natural Language Toolkit (NLTK) library and additional irrelevant terms specified in an electronic supplementary material (electronic supplementary material, table S1). To uncover the underlying themes within the literature data, we employed LDA, a robust method for topic modelling [33]. The pre-processed literature was transformed into a bag-of-words model using the Count Vectorizer technique [34]. The data were then converted to a Gensim-compatible format, generating both a dictionary and a corpus (a structured collection of processed text data), which mapped each word to a unique ID and represented each document as a bag of words.

To determine the optimal number of topics, we calculated coherence scores for different numbers of topics ranging from 2 to 20 (electronic supplementary material, figure S1). Topic coherence measures the degree of semantic similarity between high-scoring words in each topic, providing a quantitative metric for topic interpretability [35]. Higher coherence scores indicate more semantically coherent and interpretable topics. Our analysis revealed that the coherence score peaked at *n* = 15 topics (coherence score = 0.563; see electronic supplementary material, figure S1, topic coherence values), suggesting this as the optimal number of topics for our dataset. This finding aligns with previous studies suggesting that topic numbers yielding the highest coherence scores typically produce the most meaningful and interpretable results in scientific literature analysis [36].

An LDA model was then trained on the corpus using this optimal number of topics, with 15 passes to ensure thorough learning, with a fixed random state for reproducibility. The LDA results were visualized using pyLDAvis [37], which provided an interactive platform to explore the topics and their relationships. This visualization assisted in interpreting the distribution of terms within each topic and the distances between different topics in a two-dimensional space.

### 2.6. Research method extraction using a large language model

To analyse the evolution of research methods in vertebrate palaeontology over the past decade, we utilized the Qwen 2.5−72b large language model [38] for systematic method extraction. The full-text articles, previously pre-processed and verified, were input into the model to automatically extract descriptions of research techniques from each article. We developed a series of prompts to guide the model in recognizing a wide range of relevant techniques while minimizing redundancy and misclassification. Each prompt iteration was manually evaluated for accuracy, completeness and clarity by comparing the outputs of different prompts to identify the one that best matched the expected results. A sample of documents was evaluated to test various prompt configurations and the prompt yielding the most satisfactory extraction results was selected for the final analysis.

### 2.7. Method classification and standardization

To track the temporal changes in research methodologies, we established a standardized classification system. Using the Qwen 2.5−72b large language model, we performed semantic analysis to derive and standardize predefined categories of palaeontological techniques. The model identified and grouped techniques such as ‘Phylogenetic Analysis’, ‘Morphological Analysis’ and ‘High-Resolution Imaging Techniques’, ensuring a comprehensive and consistent classification of methodologies. By mapping each extracted method to these categories, we ensured consistency across the dataset, enabling temporal trend analysis and visualization of methodological shifts over the studied decade.

## 3. Results

### 3.1. Global distribution of newly defined taxa

Among the total 12 104 publications analysed, 3662 publications in vertebrate palaeontology worldwide have established 8336 new taxa, representing 30.25% of all publications in our dataset. Among these new taxa, 2812 are new genera (gen. nov.), 5049 are new species (sp. nov.), 34 are new subspecies (subsp. nov.), 412 involve new combinations (comb. nov.) and 29 are new names (nom. nov.).

Regarding the distribution among major vertebrate groups, fishes account for 29.76% (2481 new taxa), amphibians for 2.80% (233 new taxa), reptiles for 25.34% (2112 new taxa), birds for 7.39% (616 new taxa) and mammals for 34.72% (2894 new taxa). Excluding seven taxa situated at national borders and one in international waters (for which precise geographic locations could not be determined), we successfully analysed and visualized the remaining 8328 new taxa. These types of specimens are distributed across 128 countries or regions.

**Figure 1.**
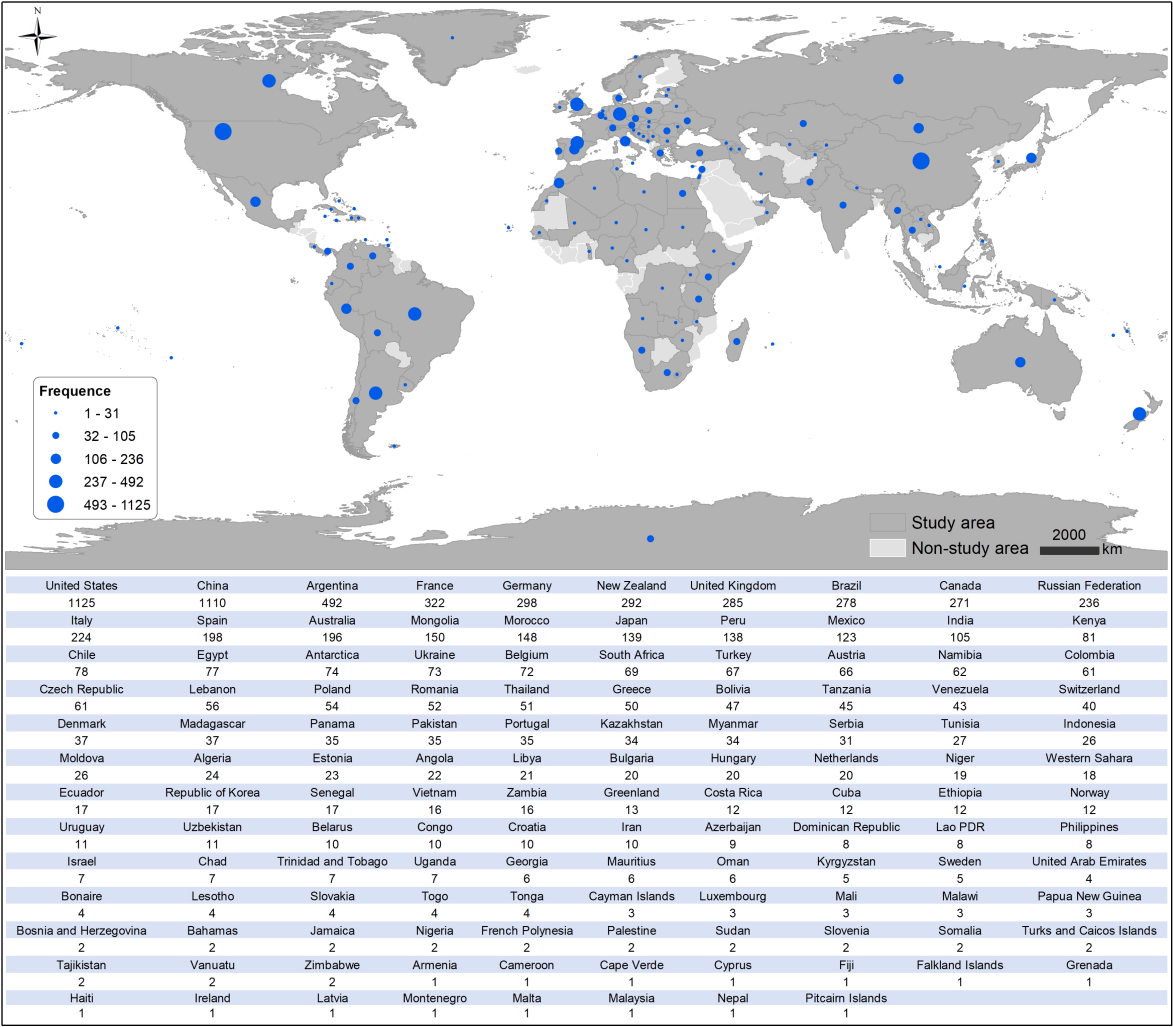
Study areas of new taxa on vertebrate palaeontology during 2014−2023.

The USA has the highest number of newly established taxa, totalling 1125 taxa (13.50%), with mammals comprising the largest group (484 taxa, 43.02%). China ranks second with 1110 taxa (13.32%), also predominantly mammals (439 taxa, 39.55%). Argentina ranks third with 492 taxa (5.90%), where reptiles are the largest group (195 taxa, 39.63%). France and Germany follow in fourth and fifth positions, with 322 taxa (3.86%) and 298 taxa (3.57%), respectively. In France, mammals are the most studied group of all new taxa (141 taxa, 43.79%), while in Germany, fishes are predominant (111 taxa, 37.25%; figure 1 and electronic supplementary material, table S2).

Further analysis revealed that monospecific genera (genera containing only one species) account for 2140 taxa, representing 25.67% of the total new taxonomic units (8336) before excluding duplicated counts. When counting these monospecific genera only once (rather than counting both the genus and its single species separately), we identified 6196 unique taxonomic units. For visualization purposes, excluding six taxa situated at national boundaries, we mapped 6190 unique taxonomic units in our regional analysis. Importantly, this methodological adjustment did not substantially affect the regional ranking of new taxonomic contributions, indicating that monospecific genera are relatively evenly distributed across global vertebrate palaeontology research (electronic supplementary material, figure S2).

### 3.2. Gender gap in vertebrate palaeontology

Among all authors, there were 4741 corresponding authors, accounting for 25.35% of the total, with one corresponding author (0.02%) for whom gender information was not available in our dataset. Additionally, there were 5242 first authors, representing 28.02% of the total, with eight first authors (0.15%) for whom gender information was not available. Over the past decade, a total of 18 705 authors contributed to vertebrate palaeontology research. Before presenting our quantitative analysis, it is important to acknowledge that while this study primarily focuses on binary gender categories due to current data collection limitations in academic publishing, we fully recognize the existence and valuable contributions of non-binary and gender-diverse individuals in vertebrate palaeontology. Their inclusion and representation are fundamental to creating an equitable academic environment. The following analysis reflects available gender information from institutional records and should be understood within these methodological constraints. The use of the Genderize tool represents a methodological choice that acknowledges gender may change over time, with our analysis capturing information available at the time of publication. This approach also respects that some individuals may be unable or unwilling to disclose their gender for various reasons. Among these, 32 authors (0.17% of the total) could not be automatically classified by the Genderize tool (probability less than 0.8) and were manually verified using telephone inquiries and institutional web searches. While most author gender information was obtained through the Genderize tool or institutional website profiles, we acknowledge that some cases remained unresolved due to unavailable biographical information or authors’ choice regarding gender disclosure, and we have analysed the data based on available gender information while recognizing these limitations. We opted for Genderize given its proven reliability in academic gender-based analyses [30], and our dual-verification approach further minimized potential classification errors.

**Figure 2.**
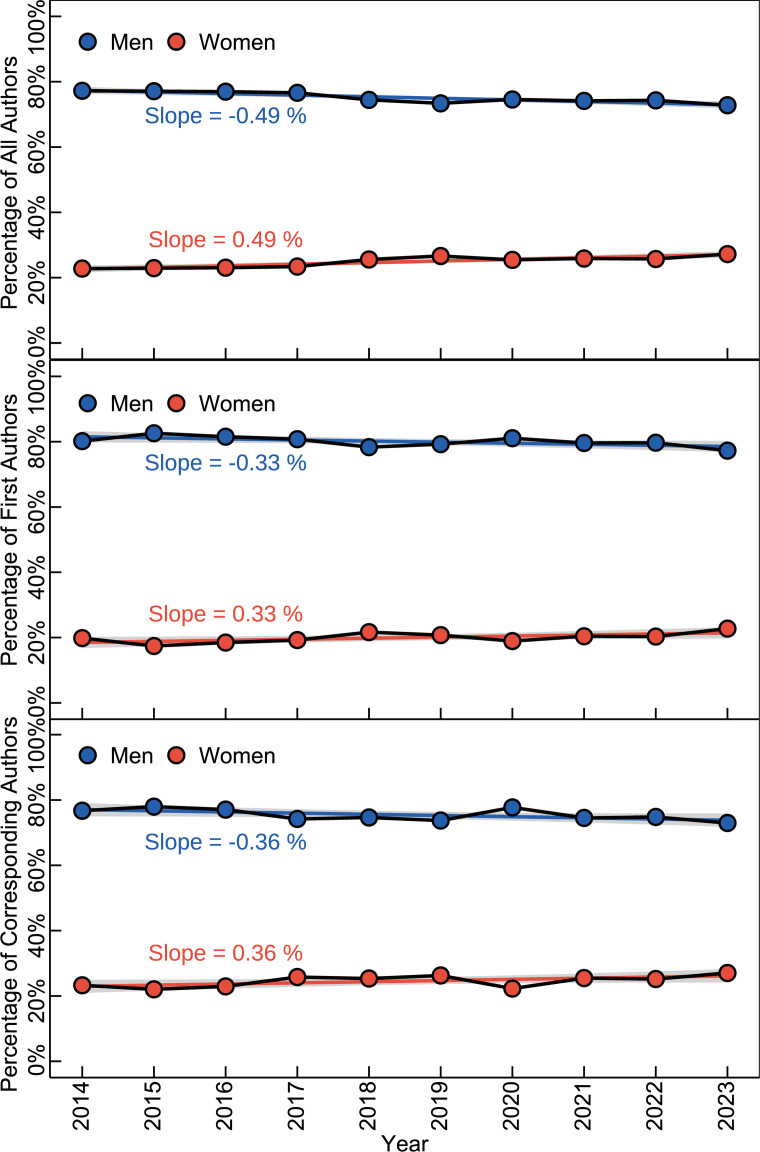
Annual change of gender ratio in vertebrate palaeontology during 2014−2023.

In all the analysed publications over the past decade, the average proportion of male authors was 67.53%, significantly higher than that of female researchers. The proportion of male first authors was 76.29%, and male corresponding authors accounted for 68.44%. From 2014 to 2023, the proportion of male authors gradually slightly declined, while the percentage of female authors increased from 26.06 to 30.87% (figure 2). Similarly, the proportion of male first authors slightly decreased from 78.48 to 75.43%, and the percentage of male corresponding authors dropped from 74.73 to 69.56% (figure 2). Moreover, the publication proportions by gender for all authors, first authors and corresponding authors are consistent with these trends (electronic supplementary material, table S3).

**Figure 3.**
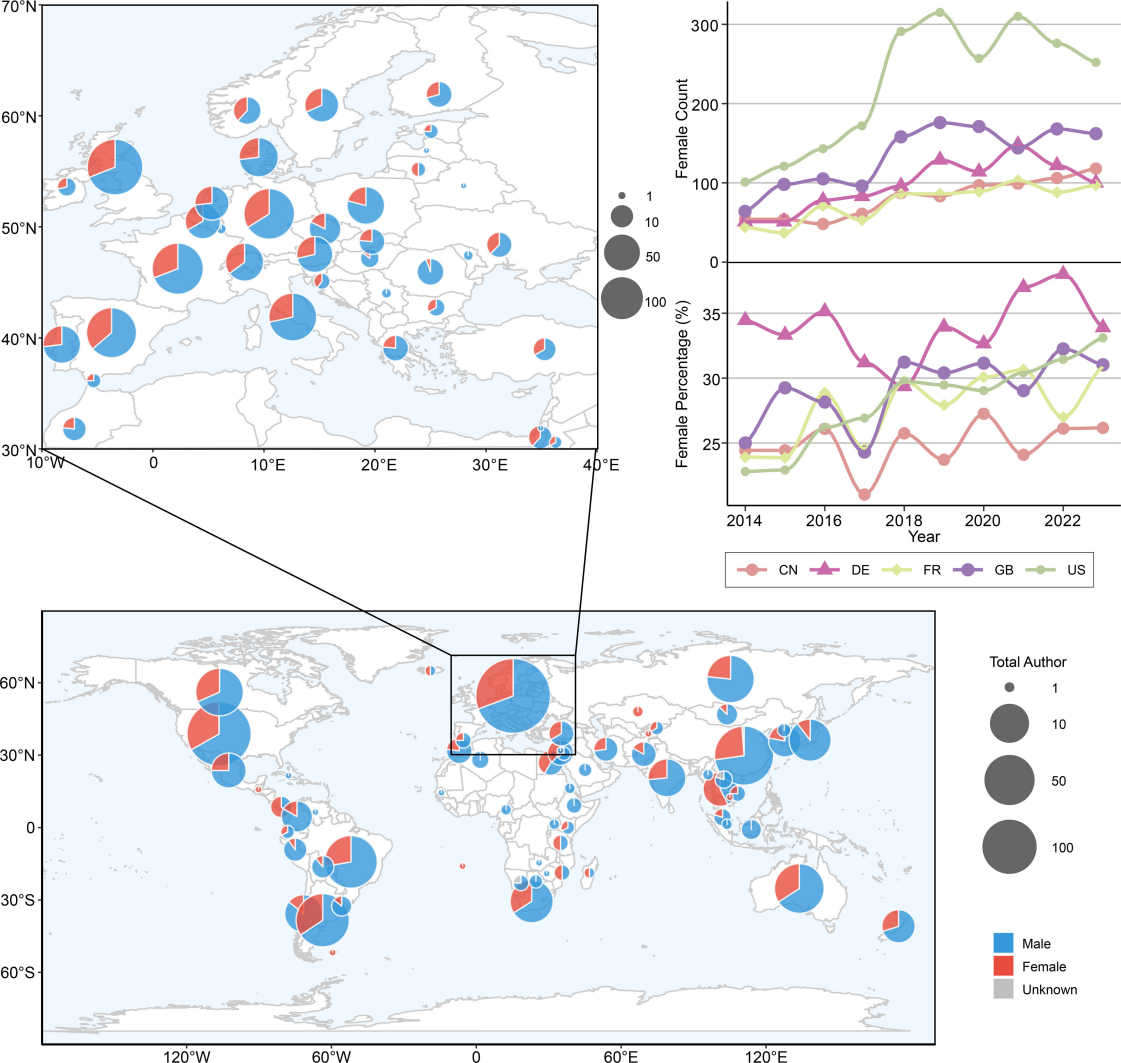
Global distribution of female scholars in vertebrate palaeontology research in 2023, displayed as the proportion of female authors by country. The pie charts illustrate regional variations, while the line graphs show the temporal trends in female scholar proportions among the top five countries by publication volume (2014−2023).

Through an analysis of author gender distribution in vertebrate palaeontology publications in 2023, countries with higher research output showed varying proportions of female authors, as illustrated by the pie charts in figure 3. Australia (34.12%), Germany (33.90%) and the USA (33.11%) showed relatively higher proportions of female representation compared with the global average. In contrast, most Asian countries showed lower proportions of female researchers, although exceptions exist. Similarly, Argentina and Brazil also showed comparable female authorship rates in 2023, with Argentina at 31.21% (132 of 423 authors) and Brazil at 27.38% (95 of 347 authors). A similar pattern is observed in the proportion of research output by female scholars globally (electronic supplementary material, table S4). Among the top five countries by publication volume, Germany has the highest proportion of female vertebrate palaeontologists (35.11%), followed by the UK (34.70%), the USA (34.41%), France (33.24%) and China (25.61%). However, when examining all countries regardless of publication volume, several nations demonstrate notably higher proportions of female participation. Among regions with research contributions, Thailand showed substantial female representation (30 female authors out of 52 total authors, 57.69%) and Croatia (14 female authors out of 25 total authors, 56.00%) in vertebrate palaeontology research. This pattern was also observed in several countries with smaller research communities: the Philippines (9 female authors out of 14 total authors, 64.29%), Angola (6 female authors out of 10 total authors, 60.00%), Lithuania (7 female authors out of 12 total authors, 58.33%) and Iceland (7 female authors out of 13 total authors, 53.85%). Single author contributions with female representation were documented from the United Arab Emirates, Bahamas, Belize, Falkland Islands (Malvinas), Cambodia, Liberia and Saint Helena, Ascension and Tristan da Cunha. Furthermore, when examining research output patterns by country, we found distinct temporal trends in female researcher proportions. As shown in the line graphs on the right side of figure 3, the proportion of female researchers in these countries has generally increased over the past decade (electronic supplementary material, table S4). Looking at the 10-year averages (2014−2023), among the top five countries by publication volume, German female authors maintained the highest proportion (31.74%), followed by the USA (26.36%), the UK (26.22%), China (25.72%) and France (25.65%). In 2023 specifically, the USA showed the highest proportion of female authors (31.52% of 403 publications), followed closely by Germany (31.00% of 210 publications), while the UK (27.01% of 316 publications), France (25.49% of 136 publications) and China (24.01% of 168 publications) showed lower proportions. Notably, the research output proportion of female authors in China and France has shown a decreasing trend over the past decade, whereas Germany, the USA and the UK have experienced a gradual increase in this metric (electronic supplementary material, table S4).

**Figure 4.**
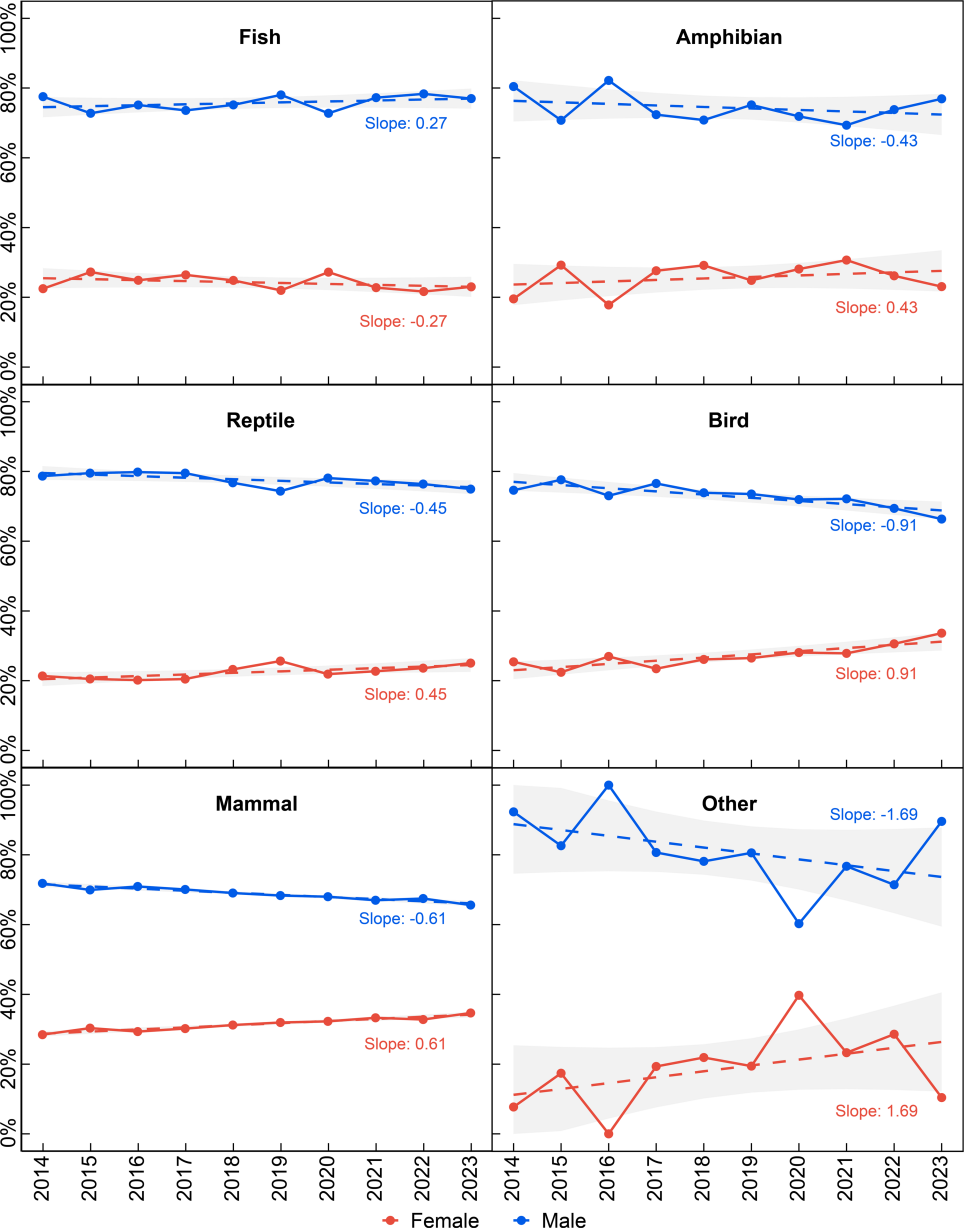
Trends in the proportion of female researchers in global vertebrate palaeontology across different taxonomic groups from 2014 to 2023.

Over the past decade (2014−2023), based on publication authorship data in electronic supplementary material, table S5, the proportion of female authors varied across different vertebrate groups in palaeontology. Mammal studies showed the highest average proportion of female authors (29.01%), followed by birds (26.73%) and amphibians (24.52%), while fishes (22.87%) and the ‘other’ group (19.92%) ranked fourth and fifth, respectively. Reptiles had the lowest proportion (19.84%). These patterns are illustrated in figure 4, which shows the temporal trends in female authorship across taxonomic groups. The proportion of female authors across different taxonomic groups showed variable patterns over the decade, as quantified by the slopes of regression lines. Birds exhibited the steepest positive trend with a slope of 0.91 percentage points per year, followed by mammals (0.61), reptiles (0.45) and amphibians (0.43). In contrast, fish studies showed a slight negative trend with a slope of −0.27 percentage points per year, indicating a gradual decrease in female participation despite the fluctuations observed in the yearly data.

### 3.3. Thematic identification through latent Dirichlet allocation analysis

**Figure 5.**
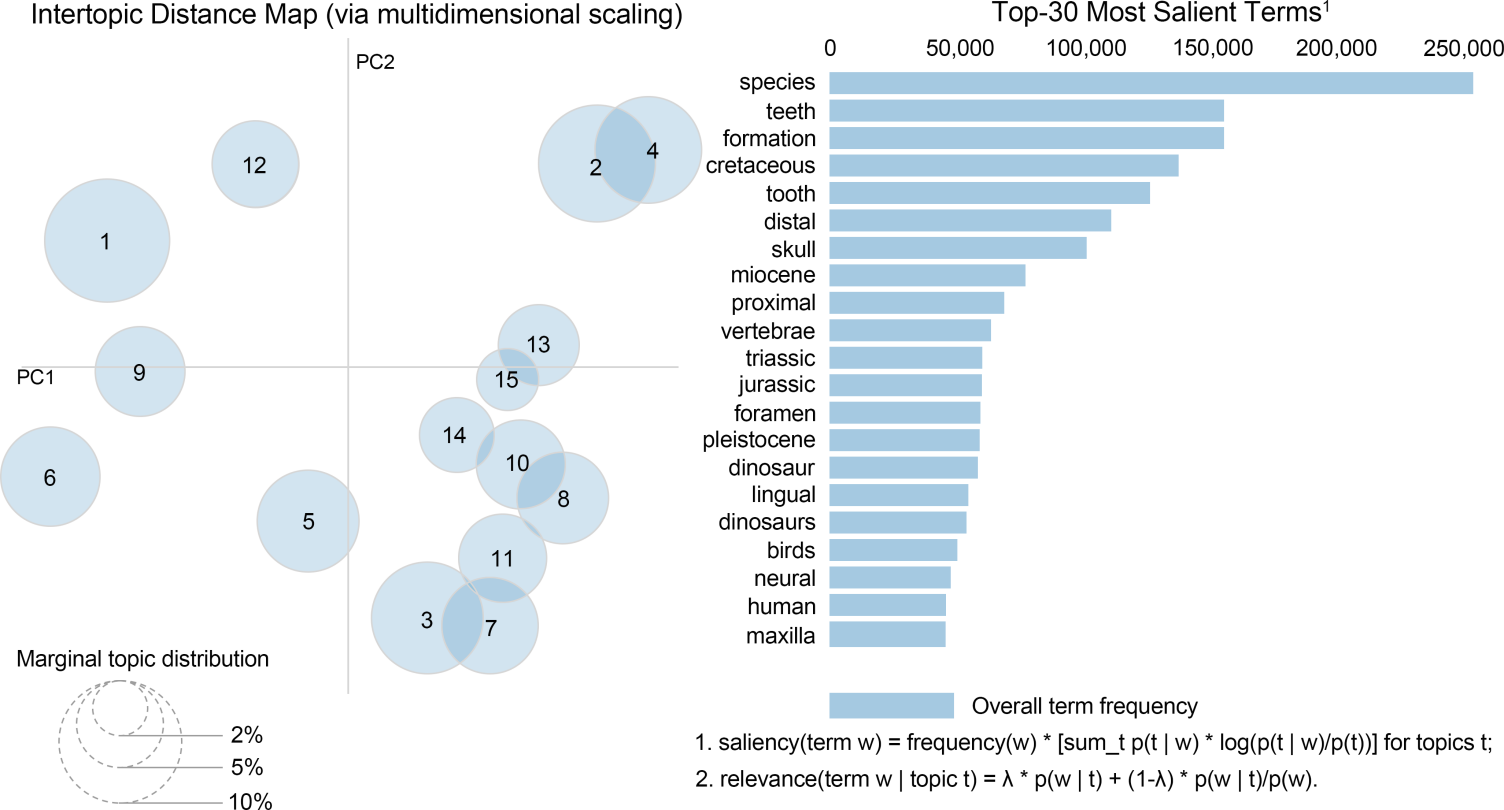
LDA analysis of top 30 most salient terms and intertopic distance map in vertebrate palaeontology research.

**Figure 6.**
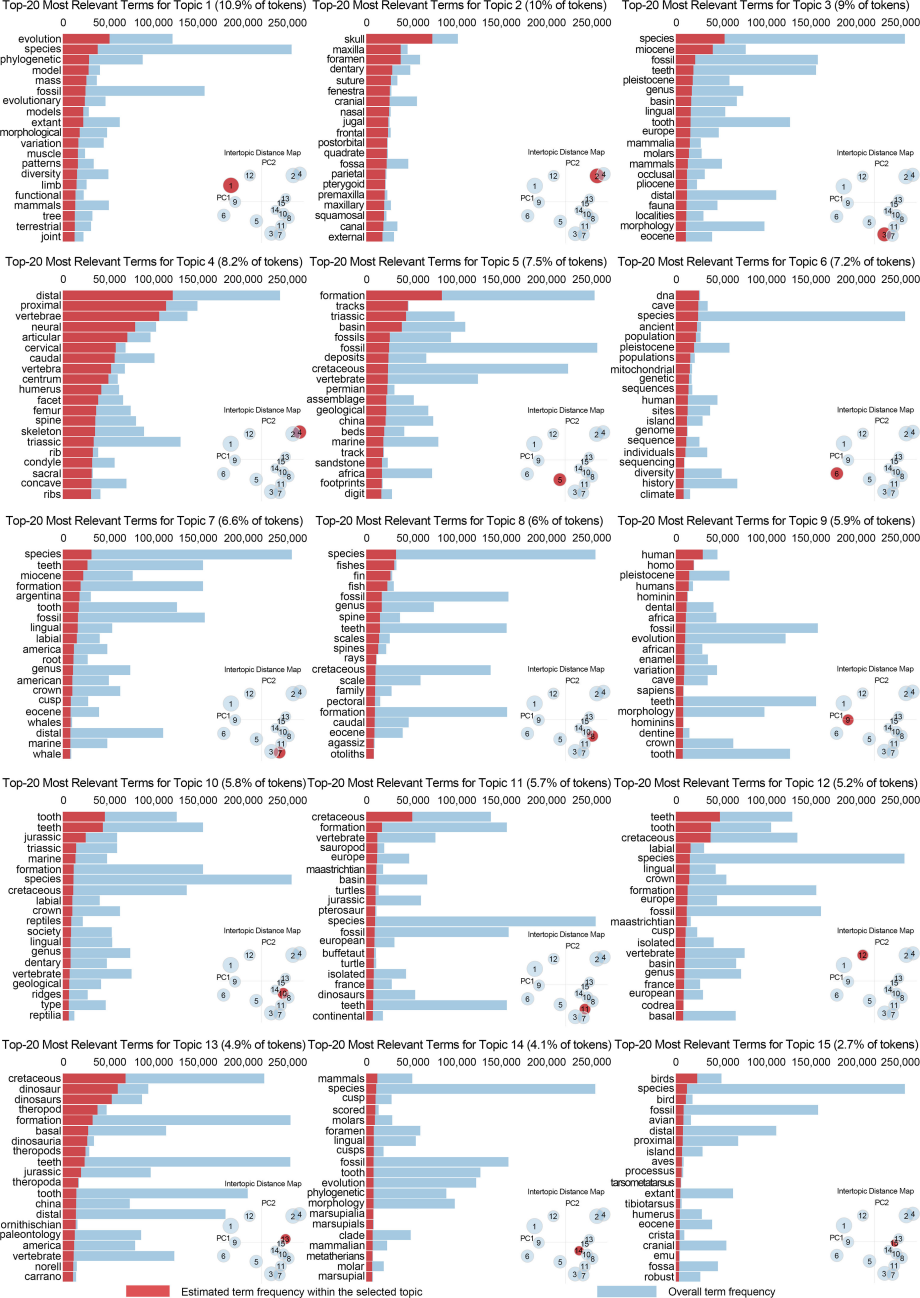
Results of the LDA analysis displaying the topics and their most relevant terms, summarizing the thematic findings.

The multidimensional scaling visualization of the LDA results (figure 5) revealed distinct spatial distribution patterns among the 15 identified topics in the two-dimensional principal component (PC) space. These topics formed several distinct clusters based on their thematic relationships, further detailed in figure 6 and electronic supplementary material, table S6.

A loose cluster formed in the left portion of the plot (figure 5) represented methodologically distinct approaches, including topics 1, 6 and 9. Topic 1 focused on evolutionary biology and phylogenetics, characterized by terms like ‘evolution’, ‘species’ and ‘phylogenetic’ (figure 6). Topic 6 concentrated on ancient DNA analysis and population genetics, featuring terms such as ‘DNA’, ‘population’ and ‘cave’ (figure 6). Topic 9 emphasized human evolution and palaeoanthropology, with key terms including ‘human’, ‘homo’ and ‘hominin’ (figure 6).

In the upper portion of the plot (figure 5), topics 2 and 4 show significant overlap, indicating strong thematic relationships in cranial and skeletal morphology. Topic 2 emphasized cranial anatomy, particularly skull morphology, with key terms including ‘skull’, ‘maxilla’ and ‘foramen’ (figure 6), while topic 4 focused on vertebral and limb morphology, characterized by terms such as ‘vertebrae’, ‘proximal’ and ‘cervical’ (figure 6).

Topics 13 and 15 form a tight cluster in the upper-right region (figure 5), representing dinosaur–bird evolution studies. Topic 13 centred on dinosaur systematics, particularly theropods and ornithischians, while topic 15 specialized in avian palaeontology and broader skeletal morphology (figure 6).

The central-right region of the plot (figure 5) contains a complex interconnected group comprising topics 3, 7, 8, 10, 11 and 14, representing various aspects of vertebrate diversity. Topics 3 and 7 show close proximity, both focusing on Cenozoic mammals but with different emphases on terrestrial and marine taxa, respectively. Topics 8 and 10 share connections through their focus on early vertebrates and dentition (figure 6), while topics 11 and 14 bridge between dinosaur and mammalian studies (figure 6).

Two topics occupy independent positions: topic 5, focusing on palaeoichnology and stratigraphy, maintaining loose connections with the central-right complex (figure 5), while topic 12, emphasizing histological studies, appears as an isolated cluster in the upper portion, reflecting its distinct methodological approach (figure 6).

**Figure 7.**
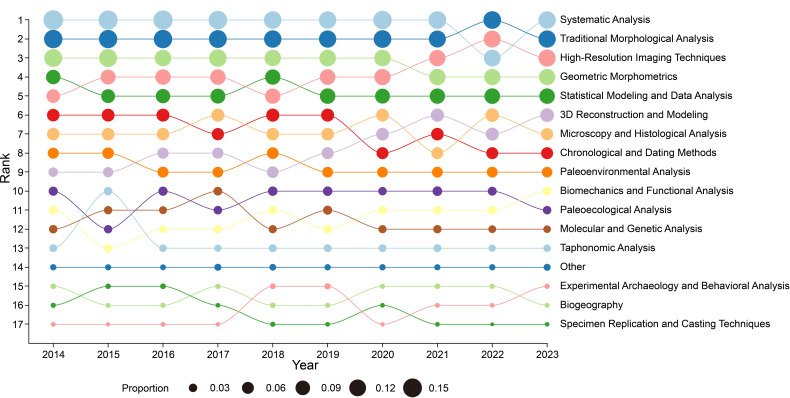
Annual variation in the use of vertebrate palaeontology research method categories (2014−2023).

The bubble chart depicts the annual changes in ranking and proportional usage of various research methods in vertebrate palaeontology from 2014 to 2023. The size of each bubble reflects the method’s relative usage, while its vertical position indicates rank compared with other methods in the respective year (figure 7).

**Figure 8.**
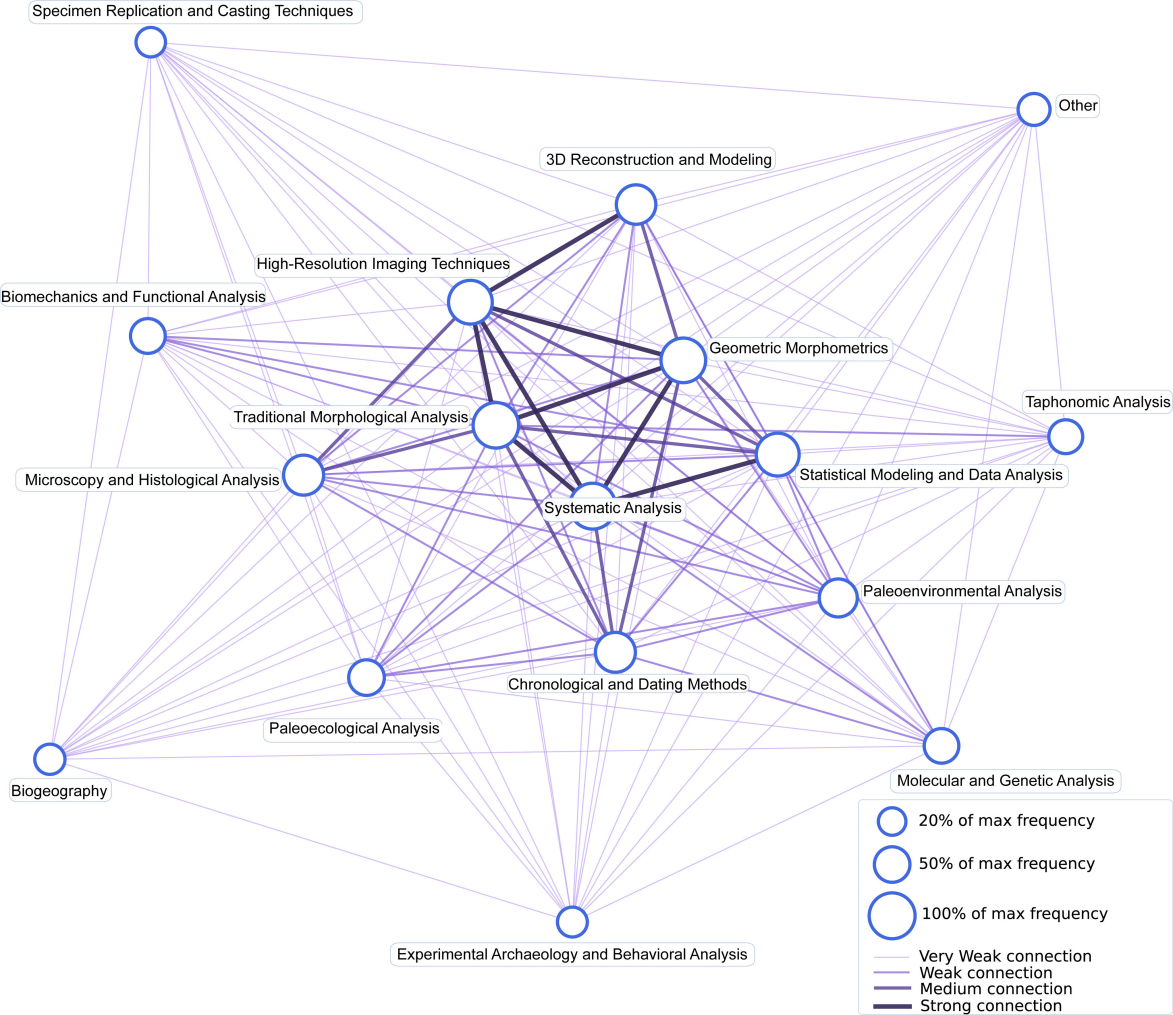
Co-occurrence network of methodological approaches in vertebrate palaeontology (2014−2023).

Edges represent co-occurrence relationships between methods, with their appearance varying by strength. The network layout was optimized using a spring layout algorithm to best display the relationships between methods. The varying edge thicknesses and colours highlight the strength of methodological connections (figure 8).

## 4. Discussion

### 4.1. Hotspot areas in vertebrate palaeontology

The significant proportion of publications defining new taxa (30.25%) demonstrates the field’s continued focus on biodiversity documentation. Among these, mammals (34.72%) and fishes (29.76%) predominate in new descriptions, followed by reptiles (25.34%), birds (7.39%) and amphibians (2.80%). This distribution pattern reflects both the preservation potential of different vertebrate groups, influenced by the robustness and nature of their remains, and their importance in understanding evolutionary transitions. The substantial number of mammalian taxa, particularly from Late Miocene and Quaternary deposits, suggests a temporal bias towards more recent geological periods. Likewise, the approximately 470 Myr presence of ‘fishes’ may explain their second ranking (adding to their aquatic lifestyle that will facilitate burial compared with terrestrial organisms).

Geographically, the USA (13.50%) and China (13.32%) emerge as leading contributors to new taxon descriptions, with both countries showing strong emphasis on mammalian palaeontology (43.02 and 39.55%, respectively). This prominence reflects their extensive territories with diverse geological formations and well-established research infrastructure, including public and private funding [2,39]. Building upon the findings of Wang *et al*. [6], Argentina’s significant contribution (5.90%), particularly in reptilian fossils (39.63%), demonstrates how regional geological heritage and tradition in the discipline shape research specialization [40].

However, the concentration of research efforts in North America, Europe and Asia (greater than 70%) versus limited contributions from Africa and Oceania (less than 10%) reveals substantial regional disparities in palaeontological research. A recent bibliometric analysis has shown that geoscience exhibits the least-developed international collaboration network among natural sciences [5]. Factors such as regional specialization, extended project durations and barriers to data sharing significantly hinder international cooperation. Addressing these challenges is essential for fostering collaboration across disciplines [5]. These disparities underscore the need for increased international collaboration to explore understudied time slices and regions with potentially rich fossil records and to balance global research efforts.

Regional disparities in research output are not solely attributable to financial resources or collaboration networks. Political instability in certain regions over recent decades has significantly reduced fieldwork, international collaboration and national funding, negatively impacting the discovery of new taxa. In such cases, research on existing museum collections represents an important alternative pathway for discovery, as has been true since the early nineteenth century, while many potentially significant specimens in these collections remain unstudied [41,42]. Intrinsic factors such as fossil resource availability, site accessibility and regional geological conditions further compound these challenges.

### 4.2. The gender representation gap in vertebrate palaeontology

Recent studies across STEM disciplines have reported similar patterns of persistent gender inequality. For instance, Huang *et al*. [43] analysed over 1.5 million academic careers globally and showed women consistently account for approximately 27% of STEM authorships—a figure very close to our findings (27.20% in 2023). They also highlighted an increasing gap in total productivity and impact despite rising female participation, underscoring structural barriers to women’s sustained advancement.

The overall trend in vertebrate palaeontology research shows concerning patterns of gender inequality as it is reported in other studies [44–48]. While there has been an increase in female representation from 26.06 to 30.87% between 2014 and 2023, with a corresponding decrease in male researchers from 73.94 to 69.13%, this change of less than 5% over a decade represents a disappointing and unacceptably slow pace of progress. At this pace, achieving gender parity could take many decades—a timeline that is entirely inconsistent with the principles of equality we should expect in the twenty-first century. Unfortunately, this pace seems to be similar in academia at discipline [26] and at global levels [45,48]. This limited progress, elsewhere documented in interdisciplinary and international collaborative research [49], underscores the urgent need for more assertive measures to address gender inequality in our field. In vertebrate palaeontology specifically, our publication data show that despite various equity initiatives (e.g. the SVP ‘Women in Vertebrate Paleontology’ mentoring scheme and the UNESCO ‘Women in Science’ fellowship), female authorship increased by only 4.81% over the past decade (from 22.78 to 27.20%). This concerning pattern is further amplified by recent findings that approximately 50% of researchers leave academia within a decade of their first publication, with female researchers showing significantly higher attrition rates [14]. Such disproportionate losses underscore the structural and cultural hurdles women encounter, ranging from implicit biases in research collaborations to limited access to leadership roles [12]. Additionally, this issue is compounded by the dual pressures of career advancement and family responsibilities, which can often feel overwhelming without systemic support [50,51]. In broader discussions of gender equality, the OECD highlights the effectiveness of policies that address structural barriers to female participation in STEM fields. These include incentivizing shared parental leave, ensuring access to affordable childcare and reforming tax-benefit systems to remove disincentives for women in employment. While addressing fundamental gender equality requires policy changes at governmental levels, the palaeontology research community can contribute by promoting inclusive practices in academic settings, such as mentorship programmes, equal collaboration opportunities and fair recognition of contributions [15]. This suggests that without substantial intervention, the field will continue to risk losing valuable talent and diverse perspectives that are essential for its advancement.

However, male researchers still dominate publication output (electronic supplementary material, table S3). The percentage of male first authors and corresponding authors remains significantly higher than that of their female counterparts, with male first authors accounting for 79.85% of the total and male corresponding authors for 75.19%. These proportions are similar to those found in other bibliometric studies of vertebrate palaeontology. In this sense, Viglino *et al*. [25] analysed the publications about Latin American extinct aquatic mammals from 1997 to 2021 and found that only 26% of the publications were led by female palaeontologists [25]. Historically, this proportion was even lower as shown in the analysis of the flagship publication of the SVP, the *Journal of Vertebrate Paleontology*. Despite making up 18% of the membership from 1981 to 1985, women with first-authored papers during this same time interval made up only 7% of authors [12]. This indicates that, despite the increasing participation of women, their representation in leading research roles remains relatively low, including in addition to publications, meeting presentations and grant success [12]. This pattern is consistent with the long-standing ‘Glass Ceiling Effect’, where women and other historically under-represented groups face persistent barriers to advancement in their academic careers, particularly in obtaining publication leadership positions (first or corresponding authors) within the scope of our bibliometric analysis [30,52]. Despite this gradual increase in female representation, male researchers continue to dominate the field in terms of both participation and publication volume. Women in vertebrate palaeontology face persistent challenges rooted in implicit biases, societal expectations and structural barriers. Historically, women were often relegated to supportive roles rather than leading positions, stemming from preconceived notions of their capabilities. As discussed by Berta & Turner [12, p. 251], women in academia were often perceived as more suited for supporting roles, based both on historical stories and contemporary interviews with female palaeontologists. This perception limited their access to leadership and decision-making roles. Historically, the earliest women vertebrate palaeontologists (1700s–1850s) studied fish and reptiles with their study of fossil mammals and other higher taxa beginning later in the nineteenth century [12]. Additionally, fieldwork—a cornerstone of palaeontology—was historically deemed unsuitable for women, further marginalizing their contributions, despite many counter-examples! The ongoing issue of sexual harassment in field and institutional settings, also highlighted in their research [12], underscores the importance of creating safe and inclusive work environments for all researchers, particularly those from historically marginalized gender identities. Whereas the participation of female researchers has increased, their representation as leading authors, including first and corresponding authors, remains relatively low.

In terms of changes in the proportion of first and corresponding authors, the increase in the proportion of women in these key roles has been slower. For instance, the proportion of female first authors rose from 19.83 to 22.74%, an increase of 2.91%, whereas the proportion of female authors overall increased from 22.78 to 27.20% (a 4.42% increase) during 2014−2023. This highlights the persistent challenges women face in obtaining leading research roles. As illustrated in figure 2 and electronic supplementary material, table S3, the proportion of female authors increased at an average rate of 0.44% per year, and the proportion of male authors decreased correspondingly. The gender gap is narrowing but at a pace that suggests achieving parity will take considerable time. These publication patterns are particularly significant as they have been demonstrated to correlate with future academic rankings [53].

To address this gender imbalance, fundamental changes are needed both within and beyond academia. Rather than simply offering professional support, what is needed is a broader transformation of academic culture, particularly in educating all members of the academic community about implicit biases and structural barriers, with special emphasis on engaging those in leadership and mentorship positions who can effect meaningful institutional change. Additionally, recognizing long-serving female researchers in vertebrate palaeontology is essential for promoting gender equity. Establishing awards, honorary titles or a recognition platform could help highlight their contributions and inspire future generations, potentially addressing some of the attrition issues discussed above. These findings underscore the critical need for comprehensive changes not only in academic institutions but also in earlier educational stages and broader society, as gender disparities in science are shaped by cultural and systemic factors that emerge well before university education. Immediate actions should address structural barriers such as unequal access to research funding, cultural biases and limited representation in leadership roles [54], and longer-term solutions must focus on transforming educational and societal attitudes towards women in science from an early age. By combining targeted mentorship opportunities with broader policy changes, such as equitable resource allocation and institutional accountability measures (e.g. enforcing codes of conduct to prevent sexual and gender-based harassment in the field and workplace), the academic community can foster an environment where female scientists and other minorities can thrive. These comprehensive measures will not only reduce the gender gap but also contribute to greater diversity and inclusivity in future vertebrate palaeontology research.

This study systematically reveals significant gender disparities in global vertebrate palaeontology over the past decade, particularly across different regions and taxonomic groups. Bibliometric studies have shown that the gender gap is country-specific and should be analysed considering different variables [30,47]. Historically developed regions have shown higher female representation in science, attributed to gender equality policies and support mechanisms [24,48], but recent political shifts in several of these nations have begun to challenge these advances. In developing countries, challenges such as cultural norms and limited educational opportunities continue to affect female participation in research [55,56]. Our analysis of the top 30 countries by publication volume (excluding those with statistically non-significant sample sizes) further supports this pattern, with developed nations (22 countries) showing slightly higher female representation (33.73%) compared with developing nations (eight countries, 30.42%). Historical data show Germany, the UK and the USA reporting higher proportions of female vertebrate palaeontologists [12,23]. Our data (2014−2023) confirm this pattern, suggesting that successful gender equality initiatives can be implemented regardless of research output volume. These patterns may reflect either inherent research interests or potential structural biases in the field that merit further investigation. However, it is important to note that recent political developments in several developed nations, including shifts in governance and policy priorities, may impact these trends and potentially threaten progress in gender equality. This emphasizes that advances in gender equality require constant vigilance and protection, as they can be vulnerable to political and social changes even in supposedly progressive contexts. Although China and France have seen an increase in the number of female researchers, their proportion of research output has declined, potentially reflecting persistent challenges such as high academic pressures and barriers to career advancement [57,58]. Regarding taxonomic research groups, our study shows that female researchers demonstrate higher participation and productivity in mammal and bird studies, with both numbers and output showing consistent annual growth. This trend may be linked to both methodological factors (e.g. integration of morphological, ecological and molecular approaches) and social network effects, where the historical presence of female researchers in these fields may create mentorship pathways that attract more women—a pattern consistent with academic homophily [59]. Recent research has shown significant gender disparities in Latin American aquatic mammal palaeontology, with women leading only 24% of publications. Notably, while in Argentina women represent the majority of specialists in this field, their contributions remain under-recognized through lower citation rates [60]. However, the declining proportion of female contributions in fish studies may indicate both resource allocation imbalances and possibly fewer established female role models in these areas.

### 4.3. Latent Dirichlet allocation analysis reveals shifts in vertebrate palaeontology research topics

Our LDA analysis of full-text vertebrate palaeontology literature from 2014 to 2023 reveals comprehensive patterns in research trends and methodological evolution. The analysis identified 15 distinct research topics, with their spatial distribution reflecting both traditional research continuity and emerging directions in the field. The intertopic distance map demonstrates three major research clusters that shape contemporary vertebrate palaeontology: a morphological studies cluster (including cranial morphology and dental studies), a taxonomic–temporal cluster (encompassing human evolution, Mesozoic vertebrates and avian palaeontology) and a methodological–contextual cluster (comprising ancient DNA, geological context, and growth and development studies). Analysis of the top 30 most salient terms reveals the dominance of anatomical features (‘skull’ and ‘teeth’), geological periods (‘Cretaceous’ and ‘Miocene’) and emerging methodological approaches (‘DNA’), the latter suggesting increasing methodological sophistication in vertebrate palaeontology research (in this case recent periods).

The evolution of research methodologies, as revealed by our co-occurrence network analysis (figure 8), confirms strong interconnections between traditional and emerging approaches. Phylogenetic analysis maintains the highest ranking (15%) throughout the decade, while high-resolution imaging techniques (9%) and traditional morphological analysis (12%) form a tightly connected methodological core. The network structure reveals three primary methodological clusters: (i) phylogenetic-morphological analysis, (ii) imaging and reconstruction techniques, and (iii) molecular–experimental methods. The temporal analysis of methodological approaches (figure 7) reveals the dynamic evolution of research methods in vertebrate palaeontology. Systematic analysis has consistently maintained the highest ranking, except for a brief period in 2022 when it was ranked third. Meanwhile, three-dimensional reconstruction and modelling techniques gained prominence by moving from rank nine to rank six over the time period.

The co-occurrence and overlapping patterns between research topics and methodological approaches demonstrate vertebrate palaeontology’s evolution towards integrated research strategies. The strong connection between ancient DNA studies (topic 12) and molecular analysis methods suggests the growing importance of interdisciplinary approaches, while the association between cranial morphology (topic 2) and high-resolution imaging techniques reflects the impact of technological advancement on traditional morphological studies. These findings align with observed trends in vertebrate palaeontology, suggesting that vertebrate palaeontology is experiencing a methodological transformation characterized by the integration of advanced analytical techniques with traditional approaches, indicating that future advances in the field may emerge from novel combinations of established and emerging research methods.

### 4.4. Research methodology evolution in vertebrate palaeontology (2014–2023)

The LDA analysis of vertebrate palaeontology literature also reveals distinct research patterns in the two-dimensional visualization through the topical distribution of the 15 identified themes. The multidimensional scaling visualization demonstrates clear clustering patterns that reflect both the fundamental structure and emerging trends in vertebrate palaeontology research; spatial distribution of topics reveals three fundamental organizational patterns. First, methodologically distinct approaches, represented by topics 1 (evolutionary biology), 6 (ancient DNA analysis) and 12 (histological studies), occupy peripheral positions in the topic space. This distribution suggests that methodological specialization tends to create distinct research communities with unique approaches and terminology, often developing as distinct research programmes before integration into mainstream palaeontological practice. Second, the strong integration between morphological studies is evident in the significant overlap between topics 2 and 4, focusing on cranial and postcranial anatomy, respectively. This integration reflects the holistic nature of morphological research in vertebrate palaeontology, where different aspects of skeletal anatomy are often studied in conjunction to understand complete organismal biology. Third, the formation of a large, interconnected complex in the central-right region (topics 3, 7, 8, 10, 11 and 14) demonstrates the integrated nature of vertebrate diversity studies. This complex reveals how research on different vertebrate groups—from early fishes to mammals—shares common methodological approaches and theoretical frameworks. The gradual transition from dinosaur–bird studies (topics 13 and 15) to this central complex suggests a continuous spectrum of research approaches across vertebrate groups, rather than sharp divisions between taxonomic specialities.

The spatial organization of topics also reveals important patterns in the development of vertebrate palaeontology as a discipline. The relative isolation of methodologically focused topics suggests that new analytical approaches often require time for validation and refinement before widespread adoption. For instance, palaeohistology (topic 12) is commonly applied across several taxa but rarely forms the basis for establishing new taxa (except in the Palaeozoic), as its focus on microstructural details does not often directly contribute to taxonomic definitions. Similarly, palaeoichnology and stratigraphy (topic 5) are strongly influenced by environmental and depositional conditions, with research often extending beyond vertebrate palaeontology into sedimentology and stratigraphy journals. The interpretation of trace fossils like footprints and faeces is particularly dependent on depositional environments and preservation conditions, highlighting the importance of environmental context in these studies. These methodological nuances highlight their specific contributions and limitations in shaping vertebrate palaeontology research. However, these methodological innovations gradually become incorporated into mainstream research practices while maintaining their distinctive characteristics. The clear clustering of topics dealing with similar taxonomic groups or anatomical regions suggests that despite increasing methodological sophistication, vertebrate palaeontology maintains strong subdisciplinary structures. These structures facilitate detailed specialist knowledge while potentially creating challenges for cross-disciplinary integration. However, the presence of bridging topics, particularly in the central complex, indicates active integration across these subdisciplinary boundaries.

Looking forward, these patterns suggest that advancing vertebrate palaeontology requires balancing specialization with integration. The LDA results indicate that while methodological innovation often occurs at the field’s periphery, successful approaches eventually become integrated into the discipline’s core practices. This suggests that future advances may emerge from both new methodological developments and novel combinations of existing approaches across subdisciplinary boundaries. The LDA analysis thus reveals vertebrate palaeontology as a field characterized by both methodological adaptability and robust traditional practices. Although histological techniques originated in pathology and anatomy rather than palaeontology, bone histological preparation methods were subsequently adopted into vertebrate palaeontology during the early twentieth century, while techniques such as computed tomography scanning from medical sciences and geometric morphometrics from biology were later adapted for this field. This integration of both native and imported methodologies, combined with clear patterns of specialization, demonstrates active cross-disciplinary exchange across subdisciplinary boundaries. This understanding provides valuable insights for guiding future research directions and fostering productive collaborations across the various field domains.

## 5. Conclusion

Our bibliometric analysis of a decade of vertebrate palaeontology research (2014−2023) provides significant insights into the recent development of the field. The study of new taxa descriptions reveals substantial regional disparities in research output, with major contributions concentrated in North America, Europe and Asia. This suggests that enhancing international collaboration and addressing intrinsic factors, such as resource availability and geological characteristics, are critical to balancing global research efforts. Demographic analysis shows a slight gradual increase in female representation from 22.78 to 27.20%, though persistent under-representation in leading research roles indicates ongoing structural barriers. Through full-text LDA analysis, we identified 15 distinct research topics, highlighting an increasingly integrated methodological landscape where traditional morphological approaches are complemented by advanced analytical techniques.

These results emphasize the need to address both geographic and demographic imbalances while promoting methodological integration in vertebrate palaeontology. They confirm the multifaceted nature of the field and underscore the potential of inclusive, interdisciplinary approaches. In alignment with UNESCO SDG 5 on gender equality, creating equitable research environments will not only help retain and elevate women in leadership positions but also foster innovative collaborations in regions where palaeontological resources remain underexplored. Strengthening support for female scientists, facilitating collaborations across socio-economically diverse regions and providing targeted funding for fieldwork can significantly enhance disciplinary productivity and global reach. Future advancements will benefit from reinforced international collaboration networks and targeted support for under-represented researchers, ensuring the continued evolution of vertebrate palaeontology as an inclusive, diverse and methodologically sophisticated discipline.

## Data Availability

The relevant data and code for the article have been archived within the Zenodo: (Original data and code—A Decade of Vertebrate Paleontology Research Global Taxa Distribution, Gender Dynamics, and Evolving Methodologies [61]. The dataset is not available for commercial use. For any related scientific research, permission must be obtained from the corresponding author (Zhaohui Pan, panzhaohui@ivpp.ac.cn). Supplementary material is available online [62].
